# Assessing the impact of protonating acid combinations in e-cigarette liquids: a randomised, crossover study on nicotine pharmacokinetics

**DOI:** 10.1038/s41598-023-37539-6

**Published:** 2023-06-29

**Authors:** Justin Frosina, Michael McEwan, James Ebajemito, Jesse Thissen, Karen Taluskie, Sarah Baxter-Wright, George Hardie

**Affiliations:** 1 British American Tobacco (Investments) Limited, Research and Development, Regents Park Road, Southampton, SO15 8TL UK; 2 Reynolds American Inc., Winston Salem, NC USA; 3 RAI Services Co., Winston Salem, NC USA

**Keywords:** Randomized controlled trials, Pharmacokinetics

## Abstract

The addition of protonating acids to e-cigarette liquid formulations (e-liquids) enhances nicotine bioavailability in e-cigarette use. However, little is known about the impact of different combinations of protonating acid on nicotine pharmacokinetics. The objectives of this study were to compare pharmacokinetics of nicotine absorption following use of a closed-system e-cigarette, containing e-liquids with two different nicotine levels and with different ratios of three common protonating acids—lactic, benzoic and levulinic. In a randomised, controlled, crossover study, nicotine pharmacokinetics and product liking were assessed for prototype e-liquids used in a Vuse e-cigarette containing either 3.5% or 5% nicotine and varying ratios of lactic, benzoic and/or levulinic acid. During an 8-day confinement period, 32 healthy adult current cigarette smokers/e-cigarette dual users used a single study e-liquid each day during 10-min fixed and ad libitum use periods after overnight nicotine abstinence. For most comparisons, C_max_ and AUC_0–60_ following both fixed and ad libitum puffing were significantly higher for e-liquids containing 5% nicotine compared with 3.5% nicotine. However, C_max_ and AUC_0–60_ were not statistically different for 5% nicotine e-liquids containing varying ratios of lactic, levulinic and benzoic acid when compared to an e-liquid containing lactic acid only. Mean scores for product liking were similar for all e-liquid formulations assessed, regardless of nicotine concentration, acid content, and whether the product was used in a fixed or ad libitum puffing regimen. While e-liquid nicotine concentration significantly affected users’ nicotine uptake, the different combinations of benzoic, levulinic and lactic acid in the e-liquids assessed had limited impact on nicotine pharmacokinetics and product liking scores.

## Introduction

The fundamental principle of tobacco harm reduction is that the health burden of combustible cigarette smoking at both the individual and population levels can be reduced by encouraging smokers to switch to nicotine and tobacco products that support combustible cigarette displacement^1^. While not risk free, smokers who switch to alternative tobacco and nicotine products reduce exposure to the smoke toxicants responsible for the morbidity and mortality associated with cigarette smoking, as compared to continued cigarette smoking^1,2^. One such alternative product is the electronic cigarette (e-cigarette), which electrically heats a liquid formulation (e-liquid) to produce an inhalable vapour^3^. Studies have shown e-cigarette vapour contains far fewer and substantially lower levels of harmful toxicants compared with cigarette smoke^2,4–6^, and research has indicated significant reductions in biomarkers of toxicant exposure when smokers switch completely to e-cigarettes^7–12^. Although not marketed for this purpose, a growing body of evidence stemming from interventional and observational studies suggest that e-cigarettes are effective in supporting smoking cessation^13–23^. Furthermore, population modeling has shown that reductions in smoking prevalence supported by exclusive e-cigarette use offer large improvements in population health by reducing smoking-related mortality^24,25^.

It has been proposed that alongside offering sensorial performance and some subjective effects similar to those experienced during combustible cigarette smoking, e-cigarettes that more closely match the nicotine delivery from combustible cigarettes are likely to have greater acceptance and are also likely to be more effective for adult smokers in providing a complete substitute for cigarette smoking^26–29^. In a recent study of adult smokers with no plans to quit smoking, participants randomised to using an e-cigarette containing 36 mg/ml nicotine were significantly more likely than those randomised to 0 mg/ml nicotine to report at least 28 days cigarette smoking abstinence after 24 weeks of use, and were also significantly more likely than those using an e-cigarette containing 8 mg/ml nicotine to report at least one or more days of cigarette smoking abstinence and more total days of cigarette smoking abstinence throughout the study^30^. Similar effects have been reported for other nicotine products, with improved nicotine delivery profiles associated with better cigarette smoking cessation support and relapse prevention^31,32^.

There are several means by which nicotine delivery to e-cigarette users can be increased, such as increasing the e-liquid nicotine concentration^28,33^, increasing the aerosol mass by applying greater power to the heating coil^28^, and/or adding protonating acids to the e-liquid^28,33^. Due to the lower volatility of protonated nicotine compared with unprotonated nicotine, e-liquids with protonated nicotine result in greater amounts of nicotine remaining in the aerosol particles until they reach the highly vascularised lung alveoli for absorption. Conversely, e-liquids with the more volatile unprotonated nicotine results in nicotine evaporation from the aerosol particles earlier and absorption therefore occurs mainly in the oral cavity and upper respiratory tract^33^. It was recently demonstrated that e-cigarette liquid protonation increases nicotine absorption, such that increasing either the nicotine content or the level of benzoic acid^28^ or lactic acid^33^ in the e-liquid increased nicotine bioavailability. Therefore, including protonating acids in e-cigarettes has the potential to more closely match nicotine delivery from combustible cigarettes, which may encourage more current smokers to use e-cigarettes as a complete substitute for combustible cigarettes.

The addition of various different protonating acids to e-liquid formulations is becoming more prevalent, with lactic, benzoic and levulinic acids recently identified as the three most commonly used among “top sellers”^34^. It is also increasingly acknowledged that this impacts nicotine delivery and pharmacokinetics from both e-cigarettes^28,33^ and other inhaled nicotine devices^27^, although little is known about the relative impact of combining different protonating acids on nicotine pharmacokinetics. The objectives therefore of the present study were to determine and compare the pharmacokinetics of nicotine absorption into the blood of cigarette smokers/e-cigarette dual users when using a closed-system e-cigarette filled with e-liquids containing various nicotine levels and with various ratios of lactic, levulinic and/or benzoic acids.

## Methods

### Study design

This was a randomised, controlled, crossover clinical study carried out at a single site operated by Simbec-Orion in Merthyr Tydfil, Wales, U.K. The study was registered on the ISRCTN registry (ISRCTN80837297). The experimental protocol was approved by the NHS Health Research Authority, Wales Research Ethics Committee 1 (reference number 20/WA/0264) prior to study commencement. The study was conducted in accordance with the protocol and under the principles of both the International Council for Harmonisation (ICH) of Technical Requirements for Pharmaceuticals for Human Use Guideline for Good Clinical Practice (GCP) E6(R2) and European Union General Data Protection Regulation (GDPR). Written informed consent was obtained from all participants prior to participation in the study and before undergoing any study procedures, including screening assessments. All participants were informed that they were free to quit smoking/using e-cigarettes and withdraw from the study, or to withdraw their consent to participate, at any time.

### Participants

Potential participants attended a screening visit, and 32 healthy male or female participants who met the inclusion criteria were enrolled in the study to ensure that a minimum of 24 participants completed the study. Participants were 21–65 years of age, inclusive, and were generally healthy as determined by clinical laboratory and physical evaluations including haematology, clinical chemistry, urinalysis, serology, urine drug and alcohol screen, medical history, physical examination, lung function tests, vital signs assessment and 12-lead ECG. All participants had at least a one year history of cigarette smoking and e-cigarette use and were current dual users of combustible cigarettes (smoking ≥ 6 mg ISO tar cigarettes and a maximum of 21 per week) and e-cigarettes (regular use of e-cigarettes with an e-liquid nicotine strength of at least 18 mg/ml). A urine cotinine threshold of ≥ 200 ng/ml was used to confirm nicotine product use status. For female participants, a negative serum pregnancy test at screening and a urine pregnancy test at check-in to the clinical site was required. Women of childbearing potential were required to use an accepted form of contraception during and for 30 days after the study.

The main exclusion criteria were pregnancy or breastfeeding (women only); self-reported non-inhalation of cigarette smoke/e-cigarette vapour; participants who did not use a flavoured e-liquid (since a flavoured e-liquid was used in the study); significant history of urticaria or asthma; blood donation of ≥ 450 ml within the three months before the participant’s first study visit, or plasma donation in the seven days prior to screening, or platelets donation in the six weeks prior to screening; participants who are planning to quit smoking or e-cigarette use; or use of any medication or substance that aids smoking cessation in the 30 days prior to the study.

### Investigational products

The products examined in this study were Vype ePod 2 (now Vuse ePod 2, referred to throughout as Vuse) closed-system e-cigarettes (Nicoventures Trading Ltd, London, UK) with prototype e-liquid formulations containing either 3.5% or 5% nicotine and various proportions of lactic, levulinic and/or benzoic acid (Table 1). Participant’s usual brand of combustible cigarettes was also assessed in this study.

**Table 1:** Prototype e-liquid nicotine and acid composition. The amounts of benzoic and levulinic acid are relative to the lactic acid amount in the sample.

Product^a^	Nicotine	Lactic acid	Benzoic acid	Levulinic acid
EPOD2.0_VFB50_1VA	5%	1	0.0	0.0
EPOD2.0_VFB35_2VB	3.5%	1	1.3	0.0
EPOD2.0_VFB35_3VE	3.5%	1	2.0	1.0
EPOD2.0_VFB50_2VH	5%	1	0.9	0.0
EPOD2.0_VFB50_3VF	5%	1	0.7	1.7
EPOD2.0_VFB50_3VJ	5%	1	1.8	0.7
EPOD2.0_VFB50_2VI	5%	1	1.2	0.0

^a^A non-commercial nectar flavour was used for all e-cigarette products.

### Study procedures

At screening, participants underwent testing to ensure that they met all inclusion and no exclusion criteria and completed tobacco and nicotine product use history questionnaires and the Fagerstrom Test for Cigarette Dependence^35^. Participants who passed screening were admitted to the clinic site the day before the first study product use session (Day-1) and were confined to the site until the last assessments at the end of the study (i.e., for a period of approximately eight days). On admission to the clinic site, participants underwent eligibility criteria review including vital signs assessments, physical examination if indicated, a test for active COVID-19 infection, and a urine pregnancy test (women only). Subsequently, participants underwent a product use session to familiarise themselves with the study e-cigarette device, where they used the product ad libitum for a minimum three-hour duration. In this session, participants would ask clinic staff for the study product, and they were provided with one of two different e-liquid formulations in an alternating manner. The 5% nicotine, EPOD2.0_VFB50_1VA e-liquid formulation was used first, followed by the 5% nicotine, EPOD2.0_VFB50_2VI e-liquid formulation. For the remainder of the familiarisation session the alternating sequence was repeated until the initiation of the first 12-h abstinence period prior to product use the following day.

Upon completion of the product familiarisation, participants began an eight-day study period in which they used one of eight assigned study products on each day. The order in which participants used the study products was set by a predetermined randomisation sequence produced using a computer generated pseudo-random permutation procedure (SAS version 9.4). The randomisation code was produced based on a Williams Latin square design for an 8 × 8 crossover study with eight sequences and four participants per sequence.

Before the product use sessions on each study day, participants were required to abstain from using any nicotine or tobacco products for a period of at least 12 h. On the day participants were randomised to smoke their usual brand cigarettes, participants smoked a single cigarette by taking ad libitum puffs over a period of 10 min. During this session, the number of puffs taken was counted. If the cigarette was finished before the end of the 10-min session no further cigarette smoking was allowed and the time the product was finished was recorded. Participants did not smoke combustible cigarettes using the fixed-puff regime, as the purpose of this regime was to isolate the impact of different protonating acid combinations contained in the prototype e-liquids on resulting PK profiles. On the days participants were randomised to use the study e-cigarettes, participants underwent two product use sessions. The first session used a “fixed-puff” regimen (one puff every 30 s for 10 min, 21 total puffs), which was followed by a six-hour period of nicotine abstinence. After this period, participants underwent a further product use session in which they took ad libitum puffs over a 10-min period, during which puffs were counted. The purpose of the ad libitum regime was to evaluate the resulting PK profiles for the prototype e-liquids and combustible cigarette products under participants’ typical usage patterns.

For all product use sessions (excluding the familiarisation session), blood samples for nicotine pharmacokinetic analysis were taken before, during and after product use. After completion of blood sampling on each study day, participants were allowed to use their own nicotine products (combustible cigarettes and/or e-cigarettes) ad libitum until 12 h prior to the next product use session. After the last study day, participants were discharged from the clinic site and a follow-up phone call was conducted with all participants 5–7 days after discharge.

### Blood sampling for nicotine pharmacokinetics

Venous blood samples (2.7 ml) were collected into lithium heparin monovette tubes at − 5 (baseline), 5, 8, 10, 15, 30, and 60 min relative to the first puff of each product use session (either fixed or ad libitum). The collected samples were processed by centrifugation at 1600*g*/3500 rpm at 4 °C for 10 min no later than 60 min after collection. Two equal aliquots (each containing approximately 1 ml) of plasma were transferred to storage tubes and stored at approximately -20 °C within 120 min after collection.

Plasma nicotine analysis was performed by liquid chromatography with tandem mass spectrometry detection (LC–MS–MS) using the instrument in turbo ionspray, positive ion Multiple Reaction Monitoring (MRM) mode. Two LC–MS–MS systems were used, AB Sciex API4000 and API5000 triple quadrupole atmospheric pressure ionisation mass spectrometers. An automated injection of samples took place using a Shimadzu SIL-20AC autosampler for each system. The API 5000 utilised a Shimadzu LC-20AD pump and the API4000 a Shimadzu LC-20AB pump. LC–MS–MS separation was performed using a Betasil-Silica-100 column using an isocratic method. The following MRM transitions were monitored: nicotine: *m/z* 163.0 → *m/z* 130.0, typical retention time 2.1 min; and nicotine − D4 (internal standard): *m/z* 167.0 → *m/z* 134.0, typical retention time 2.1 min. The measured method calibration range was 0.498–49.832 ng/ml. Samples above the upper limit of quantification (ULOQ) were diluted 1 in 10 and reanalysed. Each batch contained test samples, a reagent blank, a standard blank (i.e., a matrix blank with the internal standard omitted), and a standard zero (i.e., a matrix blank with the internal standard).

### Subjective effects assessment

At the end of each e-cigarette product use session (60 min relative to first puff), participants completed a single product liking questionnaire to evaluate the subjective effect of study product use. Answers were given by participants responding to the question “Can you tell me how much you like this nicotine product?” by making a vertical mark on a 100 mm horizontal line, with the boundaries set as “Very much” and “Not at all”. A product liking questionnaire was not completed for the usual brand cigarette product use session.

### Safety assessments

Adverse events (AEs) were recorded and monitored throughout the study from the signing of informed consent form through to the post-study follow-up visit. AEs were defined as any untoward medical occurrence in a participant to whom a product has been administered, including occurrences which are not necessarily caused by or related to that product. An AE could be any unfavorable and unintended sign, including abnormal laboratory findings considered to be clinically significant, symptom, or disease temporarily associated with the use of a study product, whether or not considered related to the product. An unexpected adverse reaction was defined as an adverse reaction where the nature or severity was not consistent with the applicable product information (e.g., documented in the Investigator’s brochure). A product-emergent adverse event (PEAE) was defined as an AE not present prior to the use of a study product or an AE already present that worsened in intensity or frequency following the use of a study product.

Serious adverse events (SAEs) were defined as any untoward medical occurrence that resulted in death, was life-threatening, required hospitalisation or prolongation of existing hospitalisation, resulted in persistent or significant disability or incapacity, was a congenital anomaly or birth defect, or was a medically significant event that required treatment/intervention to prevent one of the listed outcomes. All serious adverse events (SAEs) and AEs that had not resolved by the end of the study would be followed up by the Investigator until resolution or until the Investigator believed there would be no further change, whichever was earlier.

### Sample size and statistical methods

From previous studies, it had been observed that C_max_ least squares means (LSM) of smoking participants can reach values around 13–14 ng/ml in plasma with coefficients of variation (CVs) between 40 and 60% among the different arms. Based on these data, a sample size calculation was performed using the PROC POWER function in SAS (version 9.4; Cary, NC, USA) to assess superiority between the 5% nicotine EPOD2.0_VFB50_2VH and 5% nicotine EPOD2.0_VFB50_1VA e-liquids. This comparison assumed superiority to have a ratio between the C_max_ (maximum plasma nicotine concentration) > 1 with a ratio between means of 1.43, β = 0.2 and α = 0.05. Based on these assumptions, 24 participants completing the study was determined to be the minimum number required to successfully demonstrate superiority, providing an actual power of 0.811.

Nicotine concentration data were analysed using SAS. Prior to the calculation of derived pharmacokinetic (PK) parameters, concentration value(s) below the lower limit of quantification (LLOQ) were assigned a value of zero if the timepoint was prior to product use and LLOQ/2 otherwise.

The nicotine PK parameters C_max_ and AUC_0–60_ (area under the nicotine concentration-versus-time curve from time zero to 60 min after the start of study product use during each session) and T_max_ (time to the maximum plasma nicotine concentration during each product use session) were determined using WinNonlin Phoenix v8.2 from the individual concentration versus time data using non-compartmental methods. Demographic and PK parameters presented here are for the PK populations, which included 29–31 participants (depending on the parameter/arm) who used the randomised product, had sufficient plasma nicotine concentration–time profiles, did not use a concomitant medication which rendered the concentration profile unreliable, and did not violate the protocol in a way that may invalidate or bias the PK results. If three or more nicotine concentration values were missing or below the limit of quantification in a single use session, the participant was excluded from the PK population for that study product. Participants would also have been excluded from the PK population if the baseline (− 5 min) nicotine concentration value was higher than the post-first puff C_max_ value for that study product.

Derived PK parameters were listed and summarised for each product by gender and overall. Descriptive statistics presented were N (total study population completing each arm), n (PK set for each parameter/arm), arithmetic mean, geometric mean (with the exception of T_max_), standard deviation (SD), coefficient of variation (CV%), minimum, median and maximum. Area under the curve (AUC_0–60_) was calculated using the Linear Up-Log Down method. Following logarithmic transformation, C_max_ and AUC_0–60_ values were subjected to an analysis of covariance (ANCOVA) including fixed effects for sequence, period and product and a random effect of participant nested within sequence, with baseline %C_max_ as a covariate. Point estimates and 90%/95% confidence intervals (CI) were constructed for the comparisons of interest between each of the products using the residual mean square error obtained from the ANOVA. The point and interval estimates were back-transformed to give estimates of the ratios of the geometric LSM and corresponding 1-sided (superiority) or 2-sided 90%/95% CIs, as appropriate. In addition, estimated geometric LSM and 95% CI were presented for each product.

Statistical comparisons were performed to test the following hypotheses: the addition of different acid ratios to the e-liquid results in different plasma nicotine compared to lactic acid only; higher concentrations of nicotine in the e-liquid increases the level of plasma nicotine compared to lower concentrations; a fixed puffing protocol yields a different plasma nicotine pharmacokinetic profile compared with that of an ad libitum puffing protocol; and the plasma nicotine concentrations for each e-cigarette are not higher than those obtained from a cigarette.

For the product liking subjective effects measure, data were listed by participant and summarised by product using descriptive statistics (N, n, mean, SD, minimum, median and maximum).

## Results

### Participant demographics

32 participants met eligibility requirements and were enrolled into the study. The majority (30) of randomised participants (93.8%) completed the study according to the protocol. Two participants were withdrawn; one used study products on Days 1–6 before requesting to be withdrawn and the other participant used study products on Day 1 before requesting to be withdrawn due to an adverse event (presyncope attributed to blood withdrawal).

Basic participant demographic and tobacco/nicotine product history details are presented in Table 2; participants were all white and predominantly male (approximately 81%). All participants currently smoked less than 10 cigarettes per day, with a mean (SD) number smoked per day of 2.14 (0.588). All participants smoked factory-manufactured cigarettes with some also smoking roll-your-own cigarettes and cigarillos. All participants had been smoking for at least two years and had been using e-cigarettes for at least one year, were daily e-cigarette users, and the predominant form of e-cigarette used was a refillable tank.

**Table 2:** Demographic details and tobacco/nicotine use history of study participants.

Variable	N	%	Mean (SD)	Min	Median	Max
Age (years)	32	–	37.6 (10.64)	21	38.0	57
Sex						
Male	26	81.3	–	–	–	–
Female	6	18.7	–	–	–	–
Weight (kg)	32	–	83.83 (14.106)	57.2	84.10	111.0
Height (m)	32	–	1.766 (0.0851)	1.54	1.785	1.89
BMI (kg/m^2^)	32	–	26.775 (3.3975)	19.40	26.400	31.80
Race^a^						
White	32	100.0	–	–	–	–
Length of smoking history^a^						
2–5 years	1	3.1	–	–	–	–
5–10 years	4	12.5	–	–	–	–
10–20 years	15	46.9	–	–	–	–
> 20 years	12	37.5	–	–	–	–
Cigarettes currently smoked per day	32	–	2.14 (0.588)	1.0	2.00	3.0
Length of e-cigarette use history^a^						
1–2 years	6	18.8	–	–	–	–
2–5 years	22	68.8	–	–	–	–
5 + years	4	12.5	–	–	–	–
Type of e-cigarettes used						
Cigarette-like	1	3.1	–	–	–	–
Pre-filled cartridge	5	15.6	–	–	–	–
Refillable tank	26	81.3	–	–	–	–
Frequency of e-cigarette use^a^						
Daily	32	100.0	–	–	–	–

^a^Other potential options not shown due to values being zero. *BMI* body mass index, *SD* standard deviation, *min* minimum, *max* maximum.

### Nicotine pharmacokinetics

**Figure 1:**
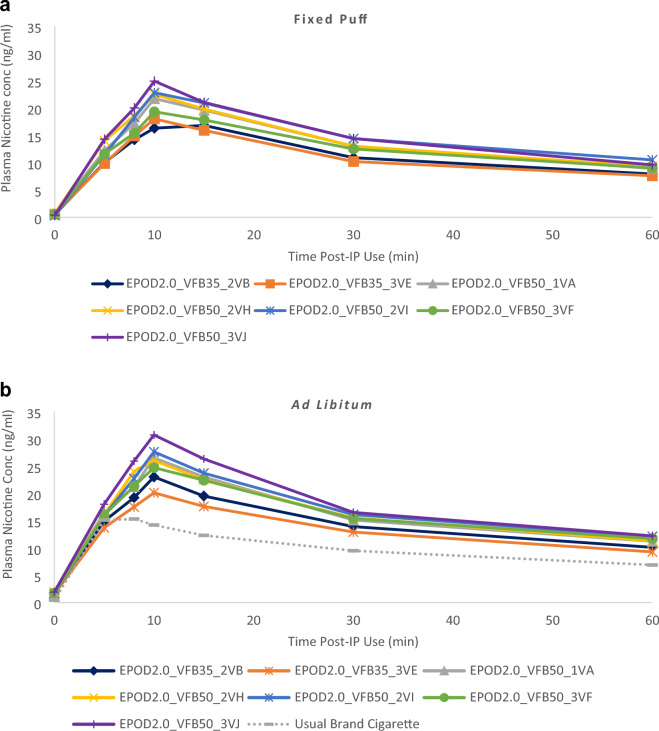
Plasma nicotine concentrations over time during fixed (**a**) and ad libitum (**b**) Puffing. Each point shows the geometric mean plasma nicotine concentration at each nominal timepoint. Error bars are not shown for clarity; for variability estimates refer to Table 3. Figures with error bars are provided in Supplementary Fig. S1. N = 29–31 (arm dependent) A: Fixed-puffing regimen; B: Ad Libitum puffing regimen.

Mean plasma nicotine concentration–time curves during fixed and ad libitum puffing of the Vuse e-cigarettes with different prototype e-liquid formulations and participants usual brand combustible cigarette (ad libitum puffing only) are shown in Fig. 1A,B, respectively. Descriptive statistics for the PK parameters C_max_, AUC_0–60_ and T_max_ are presented in Table 3. For each prototype e-liquid formulation, mean plasma nicotine concentration rose during e-cigarette use and reached a peak at the end of the 10-min fixed and ad libitum puffing sessions. For the usual brand cigarettes, mean plasma nicotine concentration rose during product use and reached a peak before the end of the 10-min ad libitum puffing session. Median T_max_ was 10 min for all e-cigarette products assessed in both puffing regimens, except for EPOD2.0_VFB35_2VB fixed puffing which had a median T_max_ of 15 min. The median T_max_ for participants usual brand combustible cigarette was 8 min (Table 3).

**Table 3:** Summary of derived nicotine pharmacokinetic parameters during fixed and ad libitum puffing.

Product	Puffing regimen	C_max_ (ng/mL)		AUC_0–60_ (min ng/mL)		T_max_ (min)		
		n	Geo mean (CV%)	n	Geo mean (CV%)	n	Median (min–max)	CV%
EPOD2.0_VFB50_1VA	Fixed	29	22.8 (55.0)	29	790 (45.0)	29	10.0 (8–30)	42.0
EPOD2.0_VFB35_3VE		30	19.1 (49.8)	28	651 (42.0)	30	10.0 (8–30)	34.8
EPOD2.0_VFB35_2VB		31	18.7 (51.4)	30	678 (46.7)	31	15.0 (8–30)	41.0
EPOD2.0_VFB50_3VF		31	20.9 (50.7)	31	758 (45.9)	31	10.0 (5–30)	49.3
EPOD2.0_VFB50_2VH		31	23.8 (49.8)	31	824 (36.8)	31	10.0 (5–30)	45.5
EPOD2.0_VFB50_3VJ		30	26.8 (68.6)	28	889 (48.4)	30	10.0 (5–30)	44.2
EPOD2.0_VFB50_2VI		29	24.6 (53.1)	29	874 (46.1)	29	10.0 (8–32)	47.6
EPOD2.0_VFB50_1VA	Ad libitum	31	29.4 (61.6)	31	969 (46.8)	31	10.0 (5–30)	34.9
EPOD2.0_VFB35_3VE		30	22.7 (75.6)	30	817 (55.3)	30	10.0 (8–30)	35.4
EPOD2.0_VFB35_2VB		31	24.5 (54.9)	31	868 (45.8)	31	10.0 (5–30)	52.3
EPOD2.0_VFB50_3VF		31	27.3 (59.3)	30	985 (45.7)	31	10.0 (5–30)	48.9
EPOD2.0_VFB50_2VH		31	28.4 (59.4)	31	973 (45.1)	31	10.0 (5–30)	39.7
EPOD2.0_VFB50_3VJ		30	33.1 (66.6)	29	1090 (50.5)	30	10.0 (8–15)	23.8
EPOD2.0_VFB50_2VI		30	29.1 (68.3)	30	1010 (51.2)	30	10.0 (8–18)	24.3
Usual Brand Cigarette		29	16.4 (32.5)	29	598 (30.3)	29	8.00 (5–10)	24.3

*C*_*max*_ maximum plasma nicotine concentration, *AUC*_*0–60*_ area under the plasma nicotine concentration–time curve between 0 and 60 min, *T*_*max*_ time of maximum plasma nicotine concentration, *Geo Mean* geometric mean, *CV% *coefficient of variation.

The PK parameters C_max_ and AUC_0–60_ during ad libitum puffing on the study e-cigarettes were all higher than those for usual brand cigarettes, regardless of the e-liquid nicotine concentration or acid composition (Table 3). For the different e-liquid formulations, C_max_ and AUC_0–60_ values were lower for the 3.5% nicotine concentration compared with the 5% nicotine concentration, regardless of acid composition or puffing regimen.

Statistical comparisons assessing the impact of the different e-liquid formulations on C_max_ and AUC_0–60_ during fixed puffing are presented in Table 4. For the e-liquids containing 5% nicotine, C_max_ and AUC_0–60_ following use of e-liquids containing lactic, levulinic and/or benzoic acid at varying levels, compared with those following use of the e-liquid containing lactic acid alone, were not significantly different (p > 0.05) across all e-liquid formulations assessed. Expectedly, for all but one comparison (C_max_ for 3.5% nicotine EPOD2.0_VFB35_3VE) C_max_ and AUC_0–60_ were significantly lower (p < 0.05) following use of the e-liquid formulations containing 3.5% nicotine compared with the e-liquid containing 5% nicotine and lactic acid only (Table 4). This impact of e-liquid nicotine concentration is demonstrated further in the statistical comparisons presented in Table 5; for the majority of the comparisons for C_max_ and for all comparisons for AUC_0–60_, increasing the e-liquid nicotine concentration from 3.5 to 5% evoked significant increases (p < 0.05) in these PK parameters.

**Table 4:** Statistical comparisons of nicotine pharmacokinetic parameters during fixed puffing—effect of acid ratios in e-liquids.

Parameter	Test product	Reference product	Geometric LSM (95% CI)		Geometric LSM ratio (90% CI) Test/Reference	Superiority test P-value
			Test product	Reference product		
C_max_	EPOD2.0_VFB35_3VE	EPOD2.0_VFB50_1VA	20.2 (16.9–24.0)	23.2 (19.5–27.7)	0.87 (0.76–0.99)	0.0662
	EPOD2.0_VFB35_2VB		19.8 (16.6–23.5)		0.85 (0.75–0.96)	**0.0348**
	EPOD2.0_VFB50_3VF		21.0 (17.6–24.9)		0.90 (0.80––1.02)	0.1773
	EPOD2.0_VFB50_2VH		23.3 (19.6–27.8)		1.01 (0.89–1.14)	0.9422
	EPOD2.0_VFB50_3VJ		25.4 (21.3–30.2)		1.09 (0.96–1.24)	0.2560
	EPOD2.0_VFB50_2VI		23.5 (19.7–28.1)		1.01 (0.89––1.15)	0.8617
AUC_0–60_	EPOD2.0_VFB35_3VE		685 (583–805)	796 (678–935)	0.86 (0.79–0.94)	**0.0063**
	EPOD2.0_VFB35_2VB		694 (591–815)		0.87 (0.80–0.95)	**0.0107**
	EPOD2.0_VFB50_3VF		760 (648–892)		0.95 (0.87–1.04)	0.3812
	EPOD2.0_VFB50_2VH		816 (695–957)		1.02 (0.94–1.12)	0.6440
	EPOD2.0_VFB50_3VJ		870 (740–1020)		1.09 (1.00–1.20)	0.1074
	EPOD2.0_VFB50_2VI		838 (713–985)		1.05 (0.96–1.15)	0.3447

*LSM* least squares means, *C*_*max*_ maximum plasma nicotine concentration in ng/ml, *AUC*_*0–60*_ area under the plasma nicotine concentration–time curve between 0 and 60 min in min ng/ml, *CI* confidence interval. Bold—statistically significant (p < 0.05).

**Table 5:** Statistical comparisons of nicotine pharmacokinetic parameters during fixed puffing—effect of nicotine level in e-liquids.

Parameter	Test product	Reference product	Geometric LSM (95% CI)		Geometric LSM ratio (95% 1-sided Lower CI) test/reference	Superiority test P-value
			Test product	Reference product		
C_max_	EPOD2.0_VFB50_1VA	EPOD2.0_VFB35_3VE	23.2 (19.5–27.7)	20.2 (16.9–24.0)	1.15 (1.02)	**0.0331**
	EPOD2.0_VFB50_3VF		21.0 (17.6–24.9)		1.04 (0.92)	0.3035
	EPOD2.0_VFB50_2VH		23.3 (19.6–27.8)		1.16 (1.02)	**0.0262**
	EPOD2.0_VFB50_3VJ		25.4 (21.3–30.2)		1.26 (1.11)	**0.0015**
	EPOD2.0_VFB50_2VI		23.5 (19.7–28.1)		1.17 (1.03)	**0.0229**
	EPOD2.0_VFB50_1VA	EPOD2.0_VFB35_2VB	23.2 (19.5–27.7)	19.8 (16.6–23.5)	1.18 (1.04)	**0.0174**
	EPOD2.0_VFB50_3VF		21.0 (17.6–24.9)		1.06 (0.94)	0.2167
	EPOD2.0_VFB50_2VH		23.3 (19.6–27.8)		1.18 (1.04)	**0.0135**
	EPOD2.0_VFB50_3VJ		25.4 (21.3–30.2)		1.28 (1.13)	**0.0006**
	EPOD2.0_VFB50_2VI		23.5 (19.7–28.1)		1.19 (1.05)	**0.0120**
AUC_0–60_	EPOD2.0_VFB50_1VA	EPOD2.0_VFB35_3VE	796 (678–935)	685 (583–805)	1.16 (1.06)	**0.0031**
	EPOD2.0_VFB50_3VF		760 (648–892)		1.11 (1.02)	**0.0264**
	EPOD2.0_VFB50_2VH		816 (695–957)		1.19 (1.09)	**0.0007**
	EPOD2.0_VFB50_3VJ		870 (740–1020)		1.27 (1.16)	** < 0.0001**
	EPOD2.0_VFB50_2VI		838 (713–985)		1.22 (1.12)	**0.0002**
	EPOD2.0_VFB50_1VA	EPOD2.0_VFB35_2VB	796 (678–935)	694 (591–815)	1.15 (1.05)	**0.0054**
	EPOD2.0_VFB50_3VF		760 (648–892)		1.10 (1.00)	**0.0418**
	EPOD2.0_VFB50_2VH		816 (695–957)		1.18 (1.08)	**0.0012**
	EPOD2.0_VFB50_3VJ		870 (740–1020)		1.25 (1.15)	** < 0.0001**
	EPOD2.0_VFB50_2VI		838 (713–985)		1.21 (1.10)	**0.0003**

*LSM* least squares means, *C*_*max*_ maximum plasma nicotine concentration in ng/ml, *AUC*_*0–60*_ area under the plasma nicotine concentration–time curve between 0 and 60 min in min ng/ml, *CI* confidence interval. Bold—statistically significant (p < 0.05).

During the ad libitum puffing sessions, participants took a similar number of puffs on each of the study e-cigarettes (Supplementary Table S1), with mean (SD) puffs ranging from 25.7 (9.98) to 28.1 (11.93). For usual brand cigarettes, participants took approximately half the number of puffs than with e-cigarettes, with a mean (SD) of 12.9 (3.52) (Supplementary Table S1). Statistical analysis of the nicotine pharmacokinetic data (Supplementary Table S2) showed that similar to fixed puffing, the effect on C_max_ and AUC_0–60_ of the addition of levulinic and/or benzoic acid at varying levels, compared with the eliquid containing lactic acid alone, was not significant (p > 0.05) across all 5% nicotine eliquid formulations assessed. C_max_ and AUC_0–60_ were significantly lower for both e-liquid formulations containing 3.5% nicotine compared with the formulation containing 5% nicotine and lactic acid only (p < 0.05; Supplementary Table S2). Further statistical comparisons of ad libitum puffing data (Supplementary Table S3) showed that, similar to fixed puffing, for the majority of the comparisons for C_max_ and for all comparisons of AUC_0–60_, increasing the e-liquid nicotine concentration from 3.5 to 5% evoked significant (p < 0.05) increases in these pharmacokinetic parameters. Additionally, C_max_ and AUC_0–60_ were significantly higher (p < 0.05) for all e-cigarette products compared with participants usual brand combustible cigarettes (Supplementary Table S4). When comparing the effect of puffing regimens, C_max_ and AUC_0–60_ were significantly higher (p < 0.05) in ad libitum compared to fixed puff use across all e-cigarette products evaluated (Supplementary Table S5).

### Subjective effects

Data for the product liking subjective effect assessment are presented in Table 6. Generally, mean scores for product liking were similar for all e-liquid formulations assessed, regardless of nicotine concentration, of acid content, and of whether the product was used in a fixed or ad libitum puffing regimen.

**Table 6:** Summary of product liking questionnaire scores.

Product	Puff regimen	n	Mean	SD	Min	Median	Max
EPOD2.0_VFB50_1VA	Fixed	31	71.4	23.78	20	81.0	98
	Ad libitum	31	68.9	26.01	6	75.0	99
EPOD2.0_VFB35_3VE	Fixed	31	73.0	20.82	22	75.0	100
	Ad libitum	31	72.6	25.13	8	78.0	100
EPOD2.0_VFB35_2VB	Fixed	31	74.5	22.92	6	81.0	99
	Ad libitum	31	76.9	22.54	8	84.0	100
EPOD2.0_VFB50_3VF	Fixed	31	74.2	18.91	12	80.0	98
	Ad libitum	31	76.6	20.83	16	86.0	100
EPOD2.0_VFB50_2VH	Fixed	31	73.1	18.34	26	74.0	100
	Ad libitum	31	72.7	20.76	25	76.0	100
EPOD2.0_VFB50_3VJ	Fixed	30	75.0	17.34	39	74.5	100
	Ad libitum	30	76.0	19.70	11	81.5	98
EPOD2.0_VFB50_2VI	Fixed	30	75.1	22.00	7	79.5	100
	Ad libitum	30	75.4	22.62	12	81.5	100

Answers were given by participants responding to the question ”Can you tell me how much you like this nicotine product?” by making a vertical mark on a 100 mm horizontal line, with the lower and upper boundaries set as “Very much” (0) and “Not at all” (100), respectively. Product satisfaction scores have been transformed (100—score) to range from 0 (not at all) to 100 (very much). *SD* standard deviation, *min* minimum, *max* maximum.

### Adverse events

There were a total of 11 PEAEs reported by 10 participants (31.3%) during the study, none of which were serious or severe and were all classed as mild or moderate events. The majority of PEAEs (six participants, 18.8%) had no reasonable possible relationship to product use. Four participants (12.5%) reported PEAEs considered to have a reasonable possible relationship to product use (one occurrence of mild dizziness and three occurrences of mild presyncope), all of which were completely resolved. It was notable that the presyncope and dizziness related events tended to occur following the fixed puffing regimen e-cigarette use. One participant was withdrawn from the study because of moderate presyncope related to blood sampling, which was completely resolved, and which was not considered to be of reasonable possible relationship to product use.

## Discussion

Within both cigarette smoke and e-cigarette vapour, the alkaloid nicotine is initially distributed in both the gas and particulate phases and deposition of nicotine mostly occurs either directly from the gas phase or following the evaporation of nicotine from the particulate phase^36,37^. The nicotine molecule itself contains basic nitrogen atoms that can be protonated, and as such three forms of nicotine (unprotonated, monoprotonated and diprotonated) are found in e-cigarette vapour^37^. Only the unprotonated form of nicotine is able to evaporate from the particulate phase and deposit in the respiratory tract, from where it enters the bloodstream. Furthermore, only the unprotonated nicotine can pass through the cellular lipid bilayers as it is lipophilic, whereas hydrophilic protonated nicotine cannot. The addition of organic acids to e-cigarette liquids alters the gas/particulate equilibrium of nicotine in the vapour resulting in a longer retention period of nicotine in the particulate phase without changing the aerosol mass of nicotine^38^. Thus, most of the inhaled nicotine is more likely to be deposited deeper in the respiratory tract^39^ where there is both a greater surface area for absorption and a greater blood flow. Through this physicochemical mechanism, acid inclusion in e-liquids increases nicotine blood absorption while reducing sensorial impact^40^, since less nicotine evaporates in the oral cavity/upper respiratory tract^28,37,41^. This may provide nicotine delivery and some sensorial experiences that more closely matches cigarette smoking and therefore improve the acceptability of e-cigarettes as a complete substitute for combustible cigarettes in current smokers.

A number of different acids have been used to protonate nicotine in e-liquids, though the most commonly used organic acids are lactic, levulinic and benzoic acid^34^. While the degree of protonation may impact nicotine uptake^28^, previous data have also suggested that the inclusion of various single organic acids in e-liquids may differentially impact nicotine absorption and heart rate (a physiological impact of nicotine)^42^. Our data suggest that nicotine uptake is not affected when using varying combinations of lactic, benzoic and levulinic acids. In accordance with this, we also found similarity in product liking when using the different prototype e-liquids which suggests that both the product liking derived from nicotine delivery as well as the sensorial impact of the aerosol were similar for the acid combinations used in the assessed e-liquids. While the e-liquid acid constituent formulation did not affect nicotine delivery, the level of nicotine inclusion appeared to be the primary driver of differences in nicotine pharmacokinetic parameters. Furthermore, greater nicotine delivery was seen during the ad libitum puffing session, which along with puff count data showing that participants took greater numbers of puffs during ad libitum use than they were allowed to during fixed use supports that user behaviour is also a driver of nicotine uptake.

It was found that nicotine absorption was higher in all e-cigarette products evaluated than the usual brand cigarettes during ad libitum use. It was also observed that participants when smoking their usual brand cigarettes took approximately half the number of puffs in the session compared to e-cigarette products. Furthermore, it was found that the T_max_ associated with cigarette use was eight minutes, before the end of the 10-min use session and earlier than the T_max_ for e-cigarette products. This indicated that the single cigarette allowed during the use session was fully completed prior to the end of the use session. This was further verified by reviewing the times the usual brand cigarette session was started and when the product was finished, which showed all participants finished the cigarette before the end of the 10-min session (data not shown). This shorter use time cannot be excluded as a contributing factor to the lower C_max_ and AUC_0–60_ parameters.

Interpretation of data from this study is subject to some limitations. Firstly, it was carried out among combustible cigarette and e-cigarette dual users in the UK and as such our findings may not be generalisable to other groups such as other geographic populations, smokers using e-cigarettes for the first time, a group whose nicotine delivery is different compared with accustomed e-cigarette users^43,44^, or smokers/e-cigarette users acclimated to different levels of nicotine. Secondly, e-cigarette use was limited to either a fixed or ad libitum use period of a brief and defined length and as such, while acute nicotine absorption was not impacted by e-liquid formulation acid content, nicotine uptake over longer periods of time cannot be inferred. Thirdly, this study only assessed the impact on nicotine pharmacokinetics of varying e-liquid levels of three organic acids; commercially-available e-liquids may contain other acids such as salicylic, malic and tartaric acid^34^ and findings from this study cannot be extrapolated to other acids or acid combinations. Lastly, since limiting participants to a single cigarette resulted in substantially less puffs and earlier T_max_ compared to the e-cigarette products in the ad libitum use session, the pharmacokinetic parameters assessed are not directly comparable in this study and duration of product use must be considered.

## Conclusion

While it has been previously shown that using single protonating acids increases nicotine pharmacokinetics, data from this study demonstrate that while the nicotine concentration and the pattern of use (fixed vs ad libitum) affected nicotine delivery, varying the ratios that lactic, benzoic and levulinic protonating organic acids were combined in e-cigarette liquids was without significant effect on nicotine pharmacokinetics and did not notably impact product liking scores. This suggests that e-cigarette products designed within the range of protonating acid combinations assessed in this study would produce a similar nicotine delivery without negatively impacting product liking as a product designed with a single protonating acid. This can be used in designing e-cigarette products that more closely match nicotine delivery and sensorial performance of combustible cigarettes, and therefore more likely to have greater acceptance and are also likely to be more effective for adult smokers in providing a complete substitute for cigarette smoking.

## Supplementary Information

Supplementary Tables.

## Data Availability

The datasets analysed during the current study are available from the corresponding author on reasonable request.
