# Supplementary material for: Assessing the impact of protonating acid combinations in e-cigarette liquids: a randomised, crossover study on nicotine pharmacokinetics
Source: Sci Rep. 2023 Jun 29;13:10563. doi: 10.1038/s41598-023-37539-6 (PMC10310785; doi:10.1038/s41598-023-37539-6)
Supplement: Supplementary file 1 — Supplementary Tables. [file 41598_2023_37539_MOESM1_ESM.pdf]

Supplementary Table S1. Puff Counts During *Ad Libitum* Puffing Session.

Product	Number of Puffs					
	n	Mean	SD	Min	Median	Max
EPOD2.0_VFB50_1VA	31	26.1	10.54	7	24.0	51
EPOD2.0_VFB35_3VE	31	27.5	12.01	12	24.0	63
EPOD2.0_VFB35_2VB	31	28.1	11.93	14	25.0	60
EPOD2.0_VFB50_3VF	31	26.8	8.92	15	26.0	56
EPOD2.0_VFB50_2VH	31	26.1	8.75	15	25.0	48
EPOD2.0_VFB50_3VJ	30	27.4	9.74	14	26.0	49
EPOD2.0_VFB50_2VI	30	25.7	9.98	12	26.5	52
Usual Brand Cigarette	31	12.9	3.52	8	12.0	22

Supplementary Table S2. Statistical Comparisons of Nicotine Pharmacokinetic Parameters During *Ad Libitum* Puffing – Effect of Acid Ratios in e-liquids.

Parameter	Test Product	Reference Product	Geometric LSM (95% CI)		Geometric LSM Ratio (90% CI) Test/Reference	Superiority Test P-value
			Test Product	Reference Product		
C_{max}	EPOD2.0_VFB35_3VE	EPOD2.0_VFB50_1VA	23.7 (19.4 - 28.9)	29.7 (24.4 - 36.2)	0.80 (0.71 - 0.89)	**0.0010**
	EPOD2.0_VFB35_2VB		24.9 (20.4 - 30.4)		0.84 (0.75 - 0.94)	**0.0086**
	EPOD2.0_VFB50_3VF		26.5 (21.7 - 32.3)		0.89 (0.80 - 0.99)	0.0844
	EPOD2.0_VFB50_2VH		28.7 (23.5 - 34.9)		0.96 (0.86 - 1.08)	0.5797
	EPOD2.0_VFB50_3VJ		30.0 (24.6 - 36.6)		1.01 (0.90 - 1.13)	0.8896
	EPOD2.0_VFB50_2VI		29.0 (23.8 - 35.3)		0.97 (0.87 - 1.09)	0.6984
AUC_{0-60}	EPOD2.0_VFB35_3VE		822 (689 - 979)	971 (816 - 1160)	0.85 (0.78 - 0.92)	**0.0016**
	EPOD2.0_VFB35_2VB		874 (733 - 1040)		0.90 (0.83 - 0.98)	**0.0405**
	EPOD2.0_VFB50_3VF		963 (808 - 1150)		0.99 (0.91 - 1.08)	0.8735
	EPOD2.0_VFB50_2VH		975 (818 - 1160)		1.00 (0.92 - 1.09)	0.9487
	EPOD2.0_VFB50_3VJ		1020 (858 - 1220)		1.05 (0.96 - 1.15)	0.3305
	EPOD2.0_VFB50_2VI		998 (837 - 1190)		1.03 (0.94 - 1.12)	0.6086

Abbreviations: LSM, least squares means; C_{max}, maximum plasma nicotine concentration in ng/ml; AUC_{0-60}, area under the plasma nicotine concentration-time curve between 0 and 60 minutes in min*ng/ml; CI, confidence interval. **Bold** – statistically significant ($p < 0.05$)

Supplementary Table S3. Statistical Comparisons of Nicotine Pharmacokinetic Parameters During *Ad Libitum* Puffing – Effect of Nicotine Level in e-liquids.

Parameter	Test Product	Reference Product	Geometric LSM (95% CI)		Geometric LSM Ratio (95% 1-sided Lower CI) Test/Reference	Superiority Test P-value
			Test Product	Reference Product		
C_{max}	EPOD2.0_VFB50_1VA	EPOD2.0_VFB35_3VE	29.7 (24.4 - 36.2)	23.7 (19.4 - 28.9)	1.25 (1.12)	**0.0005**
	EPOD2.0_VFB50_3VF		26.5 (21.7 - 32.3)		1.12 (1.00)	0.0529
	EPOD2.0_VFB50_2VH		28.7 (23.5 - 34.9)		1.21 (1.08)	**0.0028**
	EPOD2.0_VFB50_3VJ		30.0 (24.6 - 36.6)		1.27 (1.13)	**0.0004**
	EPOD2.0_VFB50_2VI		29.0 (23.8 - 35.3)		1.22 (1.09)	**0.0018**
	EPOD2.0_VFB50_1VA	EPOD2.0_VFB35_2VB	29.7 (24.4 - 36.2)	24.9 (20.4 - 30.4)	1.19 (1.07)	**0.0043**
	EPOD2.0_VFB50_3VF		26.5 (21.7 - 32.3)		1.06 (0.95)	0.1795
	EPOD2.0_VFB50_2VH		28.7 (23.5 - 34.9)		1.15 (1.03)	**0.0184**
	EPOD2.0_VFB50_3VJ		30.0 (24.6 - 36.6)		1.21 (1.08)	**0.0032**
	EPOD2.0_VFB50_2VI		29.0 (23.8 - 35.3)		1.16 (1.04)	**0.0131**
AUC_{0-60}	EPOD2.0_VFB50_1VA	EPOD2.0_VFB35_3VE	971 (816 - 1160)	822 (689 - 979)	1.18 (1.08)	**0.0008**
	EPOD2.0_VFB50_3VF		963 (808 - 1150)		1.17 (1.07)	**0.0015**
	EPOD2.0_VFB50_2VH		975 (818 - 1160)		1.19 (1.09)	**0.0006**
	EPOD2.0_VFB50_3VJ		1020 (858 - 1220)		1.24 (1.14)	**<0.0001**
	EPOD2.0_VFB50_2VI		998 (837 - 1190)		1.21 (1.11)	**0.0001**
	EPOD2.0_VFB50_1VA	EPOD2.0_VFB35_2VB	971 (816 - 1160)	874 (733 - 1040)	1.11 (1.02)	**0.0202**
	EPOD2.0_VFB50_3VF		963 (808 - 1150)		1.10 (1.01)	**0.0307**
	EPOD2.0_VFB50_2VH		975 (818 - 1160)		1.12 (1.02)	**0.0173**
	EPOD2.0_VFB50_3VJ		1020 (858 - 1220)		1.17 (1.07)	**0.0016**
	EPOD2.0_VFB50_2VI		998 (837 - 1190)		1.14 (1.05)	**0.0057**

Abbreviations: LSM, least squares means; C_{max}, maximum plasma nicotine concentration in ng/ml; AUC_{0-60}, area under the plasma nicotine concentration-time curve between 0 and 60 minutes in min*ng/ml; CI, confidence interval. **Bold** – statistically significant ($p < 0.05$)

Supplementary Table S4. Statistical Comparisons of Nicotine Pharmacokinetic Parameters During *Ad Libitum* Puffing – Comparison with combustible cigarettes.

Parameter	Test Product	Reference Product	Geometric LSMeans (95% CI)		Geometric LSMean Ratio (95% 1-sided Lower CI) Test/Reference	Superiority Test P-value
			Test Product	Reference Product		
C_{max}	EPOD2.0_VFB50_1VA	Usual Brand Cigarette	29.8 (24.5 - 36.1)	16.5 (13.5 - 20.0)	1.81 (1.60)	**<0.0001**
	EPOD2.0_VFB35_3VE		23.7 (19.5 - 28.9)		1.44 (1.27)	**<0.0001**
	EPOD2.0_VFB35_2VB		24.9 (20.5 - 30.2)		1.51 (1.34)	**<0.0001**
	EPOD2.0_VFB50_3VF		26.5 (21.8 - 32.1)		1.61 (1.42)	**<0.0001**
	EPOD2.0_VFB50_2VH		28.6 (23.6 - 34.7)		1.74 (1.54)	**<0.0001**
	EPOD2.0_VFB50_3VJ		30.0 (24.7 - 36.5)		1.82 (1.61)	**<0.0001**
	EPOD2.0_VFB50_2VI		29.0 (23.9 - 35.3)		1.76 (1.56)	**<0.0001**
AUC_{0-60}	EPOD2.0_VFB50_1VA		973 (818 - 1160)	580 (487 - 691)	1.68 (1.52)	**<0.0001**
	EPOD2.0_VFB35_3VE		826 (694 - 983)		1.42 (1.29)	**<0.0001**
	EPOD2.0_VFB35_2VB		874 (735 - 1040)		1.51 (1.37)	**<0.0001**
	EPOD2.0_VFB50_3VF		964 (810 - 1150)		1.66 (1.51)	**<0.0001**
	EPOD2.0_VFB50_2VH		974 (819 - 1160)		1.68 (1.53)	**<0.0001**
	EPOD2.0_VFB50_3VJ		1020 (856 - 1210)		1.76 (1.59)	**<0.0001**
	EPOD2.0_VFB50_2VI		1000 (841 - 1190)		1.73 (1.57)	**<0.0001**

Abbreviations: LSM, least squares means; C_{max}, maximum plasma nicotine concentration in ng/ml; AUC_{0-60}, area under the plasma nicotine concentration-time curve between 0 and 60 minutes in min*ng/ml; CI, confidence interval. **Bold** – statistically significant ($p < 0.05$)

Supplementary Table S5. Statistical Comparisons of Nicotine Pharmacokinetic Parameters – Effect of Puffing Regimen.

Product	Parameter	Geometric LSMean (95% CI)		Geometric LSMean Ratio (90% CI) Fixed Puff/ *Ad Libitum*	P-value for Comparison
		Fixed Puff	*Ad Libitum*		
EPOD2.0_VFB50_1VA	C_{max}	22.7 (18.8 - 27.4)	30.9 (25.6 - 37.4)	0.73 (0.65 - 0.83)	**<.0001**
EPOD2.0_VFB35_3VE		19.4 (16.0 - 23.4)	24.5 (20.3 - 29.6)	0.79 (0.70 - 0.90)	**0.0024**
EPOD2.0_VFB35_2VB		19.2 (15.9 - 23.2)	25.9 (21.4 - 31.2)	0.74 (0.66 - 0.84)	**<0.0001**
EPOD2.0_VFB50_3VF		20.0 (16.6 - 24.2)	27.8 (23.0 - 33.6)	0.72 (0.64 - 0.81)	**<0.0001**
EPOD2.0_VFB50_2VH		22.3 (18.5 - 26.9)	29.8 (24.7 - 36.0)	0.75 (0.66 - 0.85)	**0.0001**
EPOD2.0_VFB50_3VJ		24.0 (19.8 - 29.0)	31.8 (26.3 - 38.4)	0.75 (0.66 - 0.86)	**0.0003**
EPOD2.0_VFB50_2VI		21.9 (18.1 - 26.5)	30.3 (25.0 - 36.6)	0.72 (0.64 - 0.82)	**<0.0001**
EPOD2.0_VFB50_1VA	AUC_{0-60}	792 (667 - 939)	992 (838 - 1170)	0.80 (0.72 - 0.88)	**0.0001**
EPOD2.0_VFB35_3VE		671 (566 - 797)	840 (708 - 995)	0.80 (0.72 - 0.88)	**0.0002**
EPOD2.0_VFB35_2VB		682 (575 - 808)	890 (752 - 1050)	0.77 (0.70 - 0.84)	**<0.0001**
EPOD2.0_VFB50_3VF		742 (627 - 879)	987 (833 - 1170)	0.75 (0.68 - 0.83)	**<0.0001**
EPOD2.0_VFB50_2VH		797 (673 - 944)	994 (839 - 1180)	0.80 (0.73 - 0.88)	**0.0001**
EPOD2.0_VFB50_3VJ		842 (709 - 1000)	1050 (883 - 1240)	0.80 (0.73 - 0.89)	**0.0003**
EPOD2.0_VFB50_2VI		804 (678 - 954)	1020 (863 - 1210)	0.79 (0.71 - 0.87)	**<0.0001**

Abbreviations: LSM, least squares means; C_{max}, maximum plasma nicotine concentration in ng/ml; AUC_{0-60}, area under the plasma nicotine concentration-time curve between 0 and 60 minutes in min*ng/ml; CI, confidence interval. **Bold** – statistically significant ($p < 0.05$)

Supplementary Figure S1. Plasma Nicotine Concentrations Over Time

a

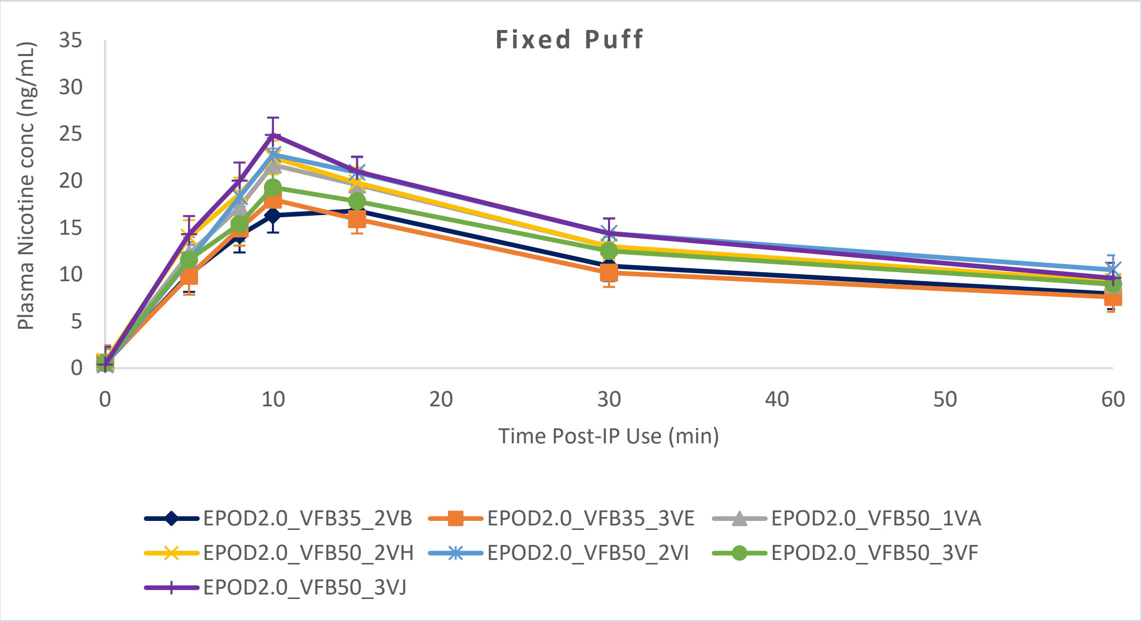

b

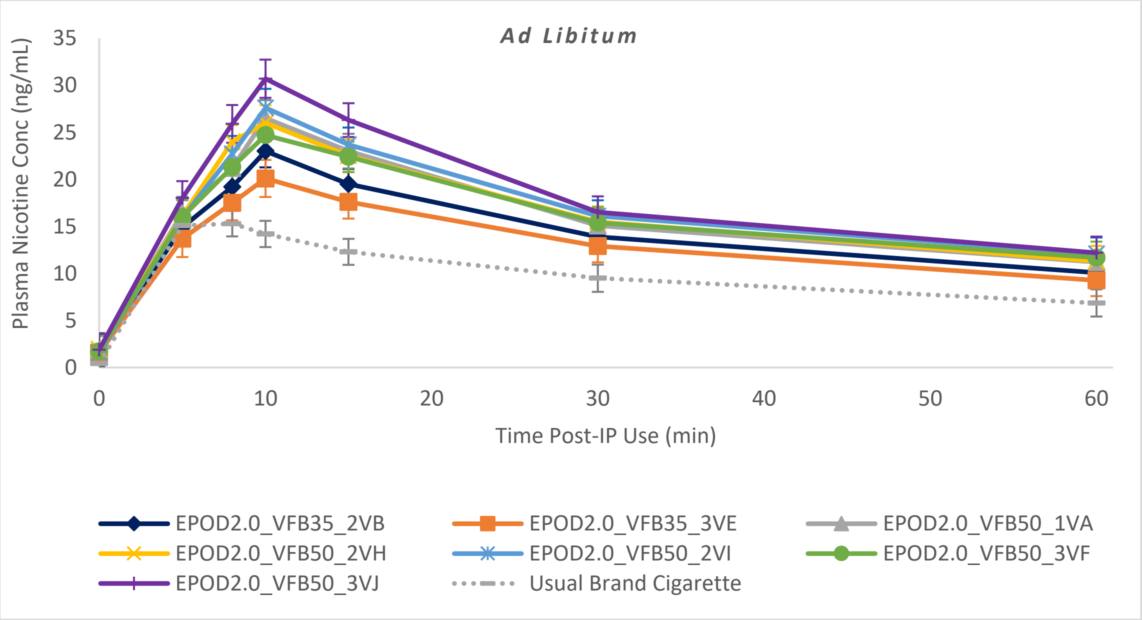

Supplementary Figure S1: Plasma Nicotine Concentrations Over Time During Fixed (a) and Ad Libitum (b) Puffing. Each point shows the geometric mean plasma nicotine concentration at each nominal timepoint with associated geometric standard deviation. The geometric standard deviation for the 0 time-point was calculated using values >LLOQ. N = 29-31 (arm dependent) A: Fixed-puffing regimen; B: Ad Libitum puffing regimen.
